# Psilocybin, From Ancestral Use to Therapeutic Potential: A Multidimensional Review of Its Pharmacological Basis and Current Perspectives

**DOI:** 10.1002/hup.70063

**Published:** 2026-09-03

**Authors:** José Norberto Vásquez‐Bonilla

**Affiliations:** ^1^ Departamento de Biotecnología Universidad Autónoma Metropolitana‐Iztapalapa Mexico City Mexico

**Keywords:** psilocin, psilocybin, psychedelic microdosing, psychedelic therapy, psychedelics

## Abstract

**Objective:**

This review critically examines psilocybin, with particular emphasis on its pharmacokinetics and pharmacodynamics, including its serotonergic, neuroplastic, and anti‐inflammatory mechanisms. It integrates historical context, preclinical and clinical evidence, and evaluates emerging therapeutic potential, safety, microdosing practices, and pharmacological interactions.

**Methods:**

A literature search was conducted in PubMed and Google Scholar using the keywords “Psilocybin,” “Psilocin,” “Psychedelic therapy,” “Psilocybe cubensis,” and “Psychedelic microdosing,” supplemented by manual searches of cross‐references. All study types were included, from randomized trials to case reports.

**Results:**

Psilocybin is metabolized into psilocin, which predominantly interacts with serotonin (5‐hydroxytryptamine, 5‐HT) receptors. These interactions contribute to enhanced synaptic plasticity, modulation of large‐scale brain networks, and regulation of immune responses via microglial activity and suppression of pro‐inflammatory cytokines. Preclinical and clinical evidence supports its efficacy in depression, anxiety, substance use disorders, neuropathic pain, epilepsy, and neuroinflammation. Psilocin also inhibits cytochrome P450 enzymes, raising concerns about drug interactions. Microdosing, though popular, remains inconclusive due to methodological limitations.

**Conclusions:**

Current findings suggest psilocybin offers sustained therapeutic effects and a favorable safety profile. However, uncertainties remain regarding long‐term safety, individualized responses, and optimal dosing. Ethical and regulatory challenges require rigorous, standardized research for responsible clinical integration.

## 1 Introduction

Since ancient times, psilocybin‐containing mushrooms have played a central role in the spiritual, healing, and cosmological practices of various Mesoamerican indigenous cultures (Strauss et al. 2022). These ceremonies, still present in communities such as the Mazateca, regarded the mushrooms as sacred entities capable of mediating spiritual healing processes. Western science rediscovered this knowledge in the mid‐20th century, initiating the first wave of psychedelic research focused on their potential therapeutic applications (Sharma et al. 2023).

However, the international prohibition of psilocybin as a controlled substance nearly halted its study for several decades (Sharma et al. 2023). From the 21st century onward, renewed interest in psychedelics has led to an accumulation of evidence regarding their safety and efficacy in treating multiple neuropsychiatric conditions (Guzmán 2015; Menon et al. 2025). At the same time, advances in neuroimaging techniques, molecular pharmacology, and preclinical models have enabled a deeper understanding of the neurobiological mechanisms underlying their effects (Shao et al. 2021; Madsen et al. 2021).

This review aims to integrate historical, ethnographic, pharmacological, and clinical findings on psilocybin, offering a critical and up‐to‐date perspective on its therapeutic potential. It explores extensively its neurobiological mechanisms, including its influence on brain connectivity, neuroinflammation, and synaptic plasticity, along with its pharmacokinetic profile and potential pharmacological interactions. Finally, microdosing is examined as an emerging approach, and the ethical and regulatory implications of psilocybin's clinical use in contemporary settings are discussed.

## 2 Historical and Cultural Perspective

### 2.1 Ancestral Use and Spiritual Context

Since pre‐Columbian times, various Indigenous cultures of Mesoamerica have used psilocybin‐containing mushrooms for ritual, healing, and spiritual purposes, integrating them into complex cosmological and health systems. In particular, the Mexica referred to them as *teonanácatl*, a Nahuatl term that can be translated as “flesh of the gods” or “sacred mushroom,” referencing both their texture and their divine character (González‐Mariscal and Sosa‐Cortés 2022; Guzmán 2015; Nichols 2020). Their use was reserved for religious ceremonies, where it was believed they facilitated access to spiritual realms and contact with deities, nature spirits, or ancestors. These practices were documented in the 16th century by friar Bernardino de Sahagún in the Florentine Codex, which describes visions, mystical states, sacred intoxication, and even physical reactions provoked by the mushrooms (Guzmán 2015; González‐Mariscal and Sosa‐Cortés 2022; Nichols 2020).

In addition to the Mexica, other cultures such as the Zapoteca, Maya, and Mixteca also developed specific ritual uses of these mushrooms, as evidenced by archaeological records, codices, and ceramics with mycological representations, which highlight the symbolic relevance of mushrooms in ceremonies associated with fertility, healing, and spiritual journeying (Afshari and Afshari 2022; Guzmán 2015).

Despite colonial efforts to eradicate their use, several Indigenous communities, including the Mazateca, Zapoteca, and Nahuatl, have preserved these traditions to the present day (González‐Mariscal and Sosa‐Cortés 2022; Rojas and Mercadillo 2024). In these contexts, mushrooms continue to be regarded as sacred agents of healing and knowledge, used to restore vital and social balance (González‐Mariscal and Sosa‐Cortés 2022; Rojas and Mercadillo 2024).

The contemporary Mazateca ritual, for example, is performed in darkness and complete silence, following strict ethical and moral codes. The mushrooms, known as *ndi‐xi‐tjo* (those who sprout), are believed to have their own voice and reveal their power only through respect and proper ceremonial preparation by the guide (Rojas and Mercadillo 2024). This worldview understands illness as a spiritual and communal imbalance, in which mushrooms act as sacred mediators.

In parallel, it has been documented that many Indigenous communities in North America share a common vision regarding the relational and non‐extractive nature of ceremonial use of sacred plants, including mushrooms, emphasizing that their power is not only in the substance itself but in intentionality, community context, and spiritual connection to nature (Tahdooahnippah 2024). This traditional perspective aligns with recent research exploring the spiritual impact of psilocybin on religious leaders from diverse faiths, including Christianity, Judaism, Islam, and Buddhism. In these controlled settings, 96% of participants ranked the experience among the most spiritually significant of their lives, reporting enduring positive changes in their ministry and a renewed sense of sacredness that mirrors the mystical insights historically documented in Indigenous traditions (Griffiths et al. 2025).

### 2.2 Origins of Modern Psilocybin Research

Knowledge of psilocybin mushrooms remained confined to traditional settings until the mid‐20th century, when Western science made its first significant contact through the experience of banker and amateur mycologist R. Gordon Wasson. In 1955, Wasson participated in a Mazatec ceremony led by the healer María Sabina in Huautla de Jiménez, Oaxaca, and documented his experience in a widely circulated article in *Life* magazine in 1957. This marked the beginning of global scientific interest in psilocybin (Nichols 2020; Sharma et al. 2023).

Wasson's publication not only sparked public curiosity but also inspired researchers such as Timothy Leary and Richard Alpert at Harvard University to conduct experiments on the psychological effects of psilocybin in humans. These trials were part of the Harvard Psilocybin Project, an exploratory initiative involving volunteer subjects that sought to expand human consciousness, although it later became entangled in ethical and political controversies (Letcher 2006; Nichols 2020).

Meanwhile, in Switzerland, chemist Albert Hofmann, known for synthesizing LSD, successfully isolated and identified the chemical structure of psilocybin and psilocin in 1958 from species of the genus *Psilocybe*. This enabled laboratory production and facilitated controlled clinical studies (Van‐Court et al. 2022; Nichols 2020; Sharma et al. 2023).

During the 1960s, various research centers, particularly in the United States and Europe, began to systematically study psilocybin in therapeutic and psychiatric contexts. Its effects were evaluated in patients with anxiety, depression, and other psychological disorders, showing preliminary benefits that were later overshadowed by its recreational use and association with the counterculture of the 1960s and 70s (Letcher 2006; Sharma et al. 2023).

### 2.3 Prohibition and the Psychedelic Renaissance

The inclusion of psilocybin as a Schedule I substance under the 1971 Convention on Psychotropic Substances drastically restricted its global use, as it was considered high‐risk and without accepted medical applications at the time (Sharma et al. 2023). This classification nearly halted scientific research into its therapeutic properties, limiting its availability in both clinical settings and traditional Indigenous contexts, where its ritual use was part of ancestral practices such as the *veladas Mazatecas* in Mexico (Rojas and Mercadillo 2024).

Strauss et al. (2022) emphasize that this international prohibition ignored preexisting ethnobotanical knowledge, including ceremonial use by Mesoamerican cultures such as the Mexica and Mazateca, who referred to the mushrooms as *teonanácatl* or “flesh of the gods.”

In the early 21st century, renewed interest in psychedelic medicine gained momentum, driven by a better understanding of psilocybin's neurobiological mechanisms, particularly its action on brain connectivity and serotonergic receptors (Sharma et al. 2023; Strauss et al. 2022). This resurgence has also been fueled by growing acceptance of psilocybin mushrooms as therapeutic tools, due to their effects on serotonergic modulation and neuroplasticity (Strauss et al. 2022).

In parallel, various United States jurisdictions began reforming their legislation. Cities like Denver, Colorado, and Oakland, California, were pioneers in decriminalizing personal use of psilocybin (Sharma et al. 2023). The most emblematic case is Oregon, which in 2021 became the first state to implement a comprehensive legal framework through the Oregon Psilocybin Services Act. This law permits the production, sale, and supervised administration of psilocybin in licensed centers under the supervision of trained facilitators. Since 2023, licenses have been issued for service centers, laboratories, and facilitators, establishing an unprecedented model of regulated access (Harder et al. 2024).

Interest has also spread to Europe. Countries like Germany and Switzerland have begun reviewing their legal frameworks, encouraged by positive clinical trial findings in treating depressive and anxiety disorders (Alyahya and Al Saleem 2025; Strauss et al. 2022). In addition to its medical implications, this resurgence has stimulated the growth of a biotechnology and pharmaceutical industry focused on psilocybin and its derivatives, attracting investment and encouraging the development of new therapeutic alternatives (Strauss et al. 2022).

Despite these advances, major barriers remain. In the United States, the federal classification of psilocybin as a Schedule I substance continues to limit access for research and treatment, requiring special permits for its handling (Sharma et al. 2023). However, recent studies have confirmed its safety profile under controlled conditions, with mostly mild and transient adverse events such as nausea, anxiety, and headache, along with sustained therapeutic effects, particularly in patients with treatment‐resistant depression, end‐of‐life anxiety, and substance use disorders (Dawood‐Hristova and Pérez‐Jover 2023; Orzechowski et al. 2025; Rojas and Mercadillo 2024; Turek 2023). This growing body of evidence has fueled a gradual shift in the social and regulatory perception of its medical use, positioning psilocybin as an emerging therapeutic alternative that is effective and has a favorable safety profile (Gattuso et al. 2024; Strauss et al. 2022; Turek 2023).

## 3 Pharmacology

### 3.1 Pharmacokinetics

**FIGURE 1:**
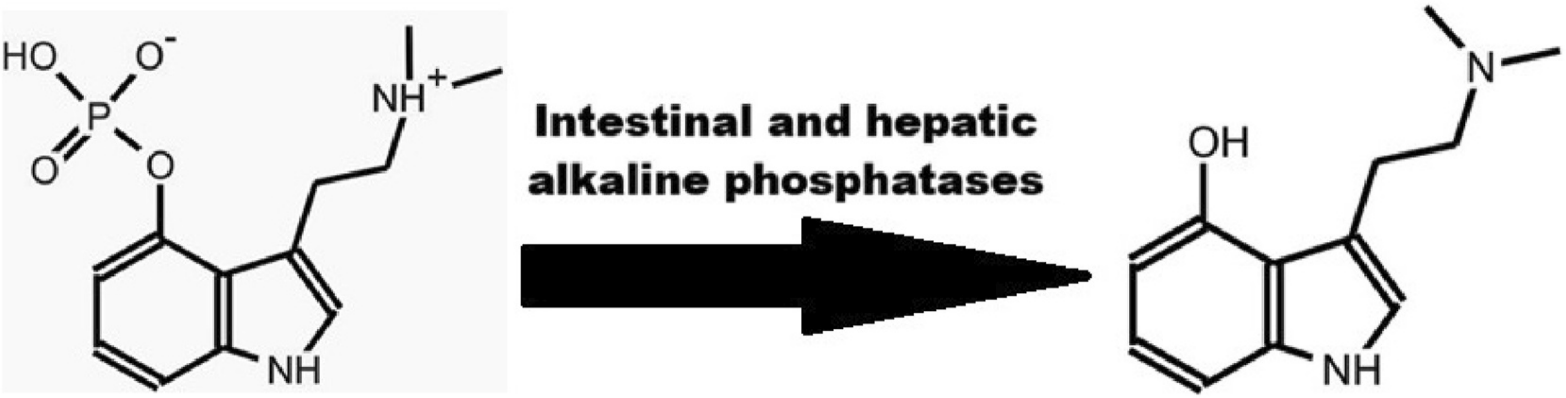
Conversion of psilocybin to psilocin via intestinal and hepatic alkaline phosphatases.

Psilocybin is a prodrug that, after oral administration, is rapidly dephosphorylated in the gastrointestinal tract and liver to produce its active form, psilocin (4‐hydroxy‐N,N‐dimethyltryptamine), which is responsible for the psychoactive and therapeutic effects observed in humans (Thaoboonruang et al. 2025; Thomann et al. 2024). This conversion occurs through enzymes such as intestinal and hepatic alkaline phosphatases (Figure 1), and virtually no unmetabolized psilocybin is detected in systemic circulation (Thaoboonruang et al. 2025).

Once absorbed, psilocin reaches peak plasma concentrations (*T*_max_) between 1.8 and 4 hours, with an elimination half‐life ranging from 1.5 to 4 hours depending on factors such as dosage, physiological status of the patient, and route of administration (Meshkat et al. 2025). Its oral bioavailability is estimated at approximately 50%–53%, and it exhibits a wide volume of distribution, ranging from 277 ± 92–1016 L, indicating substantial distribution into tissues, including the central nervous system (Meshkat et al. 2025).

Several studies have confirmed that cytochrome P450 enzyme activity also plays a relevant role in psilocin metabolism. In particular, the CYP2D6 and CYP3A4 isoforms participate in its oxidative biotransformation, and additional metabolites such as norpsilocin have been identified (Thomann et al. 2024). However, in addition to being a substrate, psilocin can act as an inhibitor of these enzymes. In an in vitro study, psilocin showed potent inhibition of CYP3A4 (IC_50_ = 2.12 μM), CYP2D6 (IC_50_ = 11.89 μM), CYP2A6 (IC_50_ = 0.99 μM), and CYP2B6 (IC_50_ = 4.05 μM), while psilocybin primarily inhibited CYP3A4 (IC_50_ = 49.43 μM) (Brito‐da‐Costa et al. 2023). These results suggest a potential risk of drug interactions, particularly in patients receiving concomitant treatments that depend on these isoenzymes.

In phase II metabolism, psilocin is predominantly conjugated via glucuronidation by UDP‐glucuronosyltransferase enzymes UGT1A9 (in the liver) and UGT1A10 (in the intestine), forming psilocin‐O‐glucuronide, one of the major metabolites detected in plasma and urine. This conjugative pathway occurs in parallel with oxidative phase I degradation, where psilocin is transformed into 4‐hydroxyindole‐3‐acetaldehyde (4‐HIA) by monoamine oxidase A (MAO‐A), and subsequently oxidized to 4‐hydroxyindole‐3‐acetic acid (4‐HIAA) via aldehyde dehydrogenase (ALDH) and aldehyde oxidase (AO) (Chen et al. 2025; Thomann et al. 2024). A significant proportion of the administered dose is excreted as psilocin‐O‐glucuronide in urine within the first 24 hours, with plasma concentrations of conjugated psilocin reaching approximately four times those of free psilocin. Additionally, about 33% of the dose is excreted as 4‐HIAA, another major metabolite (Thomann et al. 2024).

Finally, psilocin exhibits high systemic clearance, with values that may exceed hepatic blood flow (90 L/h), implying a significant contribution of extrahepatic metabolism. In this context, MAO‐A is identified as the principal enzyme involved, mediating approximately 80.9% of total hepatic clearance of psilocin, with an estimated rate of 158.74 mL/min/kg. Psilocin is converted into the intermediate metabolite 4‐HIA, which is then further metabolized by ALDH and AO into 4‐HIAA, one of the primary inactive metabolites found in the main elimination pathway: urine (Chen et al. 2025; Thomann et al. 2024).

Given the limited gastrointestinal stability and prolonged systemic exposure of psilocin following oral psilocybin administration, new psilocin‐derived prodrugs with modifications at the 4‐OH position of the indole ring have been proposed to improve its pharmacokinetic profile. Some of these compounds have demonstrated greater stability in aqueous media, improved intestinal absorption, and a modulated release of psilocin, achieving plasma levels comparable to those of psilocybin with a more controlled duration of the psychedelic effect (Raithatha et al. 2024).

### 3.2 Cytochrome P450 (CYP450) Inhibition and Pharmacological Interactions

Psilocybin is rapidly metabolized into its active form, psilocin, which has demonstrated in vitro the ability to inhibit several cytochrome P450 isoforms, including CYP3A4, CYP2D6, CYP2A6, and CYP2B6 (Brito‐da‐Costa et al. 2023). This inhibition could affect the metabolism of coadministered drugs that rely on these isoforms, although the clinical relevance of this phenomenon still requires validation through in vivo studies.

Additionally, the interaction between psilocybin and selective serotonin reuptake inhibitors (SSRIs) such as fluoxetine, sertraline, and citalopram may increase the risk of adverse effects due to interference at the serotonergic system level, including potential manifestations of serotonergic toxicity, cognitive alterations, and autonomic dysfunction (Kimberly et al. 2024).

Both observational evidence and systematic reviews have indicated that the coadministration of psilocybin with SSRIs can significantly attenuate the intensity of its psychedelic effects, possibly due to interference in serotonergic signaling (Kimberly et al. 2024; McDowell et al. 2026; Sarparast et al. 2022). Similarly, the use of benzodiazepines such as alprazolam presents a distinct challenge; while they may preserve the subjective psychedelic experience, they negatively affect memory and subsequent integration, potentially limiting the long‐term therapeutic benefits (McDowell et al. 2026). Furthermore, although infrequent, this combination may increase the risk of adverse events, including symptoms consistent with serotonin syndrome (Kimberly et al. 2024; Sarparast et al. 2022).

Psilocin has been shown in vitro to inhibit the CYP3A4 enzyme, which could prolong the half‐life of drugs metabolized by this enzyme, such as anxiolytics and antipsychotics, thereby increasing the risk of adverse effects including excessive sedation and clinical complications in combined treatments (Brito‐da‐Costa et al. 2023; Meshkat et al. 2025; Thomann et al. 2024). Therefore, careful clinical management is recommended, particularly with drugs that have a narrow therapeutic index or depend on hepatic metabolism for their elimination.

### 3.3 Pharmacodynamics

Following its dephosphorylation, psilocin predominantly interacts with serotonin (5‐hydroxytryptamine, 5‐HT) receptors, such as 5‐HT2A, 5‐HT1A, and 5‐HT4, acting as an agonist (Balabandian et al. 2025; Hakami et al. 2023; Neumann et al. 2024). At the 5‐HT2A receptors, psilocin shows greater binding affinity than serotonin itself due to its tertiary amine and specific receptor interactions (Hakami et al. 2023). Activation of this receptor induces a conformational change that affects the Gαq subunit of G proteins, ultimately triggering transcription factors that promote the synthesis of proteins related to neuronal plasticity and dendritic growth. These intracellular and network‐level effects have been linked to improvements in cognitive flexibility, synaptic restructuring, short‐term memory consolidation, reduced neuroinflammation, enhanced mood (less anxiety and depression), and slowed cognitive decline (Deco et al. 2024; Nichols 2020; Shah et al. 2025; Viohl et al. 2025; Zheng et al. 2024).

Orzechowski et al. (2025) further highlight that psilocin increases glutamate release in the prefrontal cortex, activating *α*‐amino‐3‐hydroxy‐5‐methyl‐4‐isoxazolepropionic acid (AMPA) receptors and stimulating the esecretion of neurotrophic factors like brain‐derived neurotrophic factor (BDNF) and the mechanistic target of rapamycin (mTOR) pathway, thereby enhancing synaptic plasticity and the formation of new neuronal connections. In non‐neuronal tissues such as the skin, activation of the 5‐HT2A receptor reduces inflammation, showing antipruritic effects (Afrooghe et al. 2025).

Functional connectivity between regions such as the prefrontal cortex and anterior cingulate cortex also increases, improving neuronal communication and supporting the integration of sensory and emotional information (Madsen et al. 2021; Nichols 2020). Additionally, psilocin induces a significant increase in c‐Fos expression in the paraventricular nucleus (PVN) of the hypothalamus, a region involved in neuroendocrine stress responses, suggesting a potential role of PVN activation in the therapeutic modulation of stress‐related behaviors (Effinger et al. 2024). In parallel, psilocin induces rapid and sustained synaptic remodeling in the medial frontal cortex, marked by increased dendritic spine density and size without signs of neurotoxicity in vulnerable areas such as the retrosplenial cortex, unlike the neurotoxicity reported with ketamine in similar regions. These structural changes emerge within 24 hours, persist for at least 1 month in preclinical models (Shao et al. 2021).

According to Orzechowski et al. (2025), these new synaptic connections are complemented by serotonergic effects mediated by psilocin, which foster deep introspection and reorganization of cognitive patterns, facilitating long‐lasting changes in personality and behavior. These processes are associated with enhanced emotional processing and introspective capacity, which may underlie the therapeutic effects observed in depression and anxiety disorders (Meccia et al. 2023; Nichols 2020; Shah et al. 2025). Such therapeutic effects can last for weeks or months after just one or a few doses of psilocin, due to the combination of synaptic plasticity and meaningful introspective experiences, reducing the need for chronic medication and without significant side effects compared to conventional antidepressants (Orzechowski et al. 2025; Pawlicki et al. 2024; Shah et al. 2025). In fact, adverse effects associated with psilocin are generally mild and transient, including headache, dizziness, nausea, diarrhea, fatigue, insomnia, and disturbances in sleep regulation and architecture, as well as perceptual phenomena known as flashbacks. These effects do not compromise safety or significantly interfere with daily functioning (Menon et al. 2025; Müller et al. 2022; Orzechowski et al. 2025; Thomas et al. 2022).

More serious contraindications apply to individuals with cardiovascular disease, as psilocybin can cause transient but significant increases in blood pressure, this is partly due to activation of 5‐HT4 receptors expressed in cardiac tissue, where psilocybin acts as a partial agonist, inducing inotropic and chronotropic effects observed in vivo (Neumann et al. 2024). These findings raise concern about potential cardiovascular effects linked to therapeutic or recreational psilocin use, especially in patients with heart disease or hypertension (Neumann et al. 2024). Its use is also discouraged in individuals with a history of psychotic disorders or psychiatric vulnerability, due to risks of emotional destabilization, suicidal ideation, or exacerbation of psychotic symptoms (Orzechowski et al. 2025).

On the other hand, psilocin demonstrated anticonvulsant effects, as evidenced by its evaluation in behavioral seizure models such as the pentylenetetrazole test for clonic seizures and the maximal electroshock test for generalized tonic‐clonic seizures, as well as through electrophysiological recordings in mice. These effects appear to be mediated through the involvement of the kynurenine pathway, opioidergic and nitrergic systems, and cannabinoid receptors (Balabandian et al. 2025). Orzechowski et al. (2025) reported that this effect complements psilocin‐induced modulation of glutamate and dopamine, particularly in the prefrontal cortex, promoting controlled glutamate release, AMPA receptor activation, and intracellular signaling via BDNF and the mTOR pathway. In addition to dopamine and glutamate, low doses of psilocybin stimulate the release of noradrenaline, serotonin, GABA, and acetylcholine, which correlates with anxiolytic effects and a transient modulation of the hypothalamic‐pituitary‐adrenal axis via corticosterone (Bysiek et al. 2025). These pathways enhance synaptic plasticity, supporting both immediate emotional regulation and long‐term neural connectivity. This may explain not only the reduction in anxiety but also the sustained clinical improvements linked to enhanced introspection, cognitive restructuring, and positive personality changes (Orzechowski et al. 2025; Singer et al. 2024). Notably, these clinical benefits occur without hallucinogenic activity or oxidative DNA damage, suggesting that low‐dose psilocybin offers a safer therapeutic profile with fewer adverse effects than other psychotropic compounds such as ketamine and 3,4‐methylenedioxymethamphetamine (MDMA) (Bysiek et al. 2025).

## 4 Therapeutic Applications

### 4.1 Neuroprotection and Inflammation

The therapeutic potential of psilocin extends beyond psychological outcomes, deeply engaging neuroprotective pathways and inflammatory response modulation within the central nervous systems. In the context of neurological stability, the anticonvulsant activity of psilocin involves an indirect modulation of the endocannabinoid system, rather than direct receptor binding. Experimental models show that its protective effects against seizures are significantly diminished by the CB1 receptor antagonist AM‐251, highlighting a functional dependency on the CB1 pathway (Balabandian et al. 2025). This indirect mechanism is associated with the upregulation of 5‐HT1A receptors and a subsequent downregulation of CB1 receptor expression and the indoleamine 2,3‐dioxygenase enzyme, further involving the kynurenic acid pathway, as well as the opioid and nitrergic systems (Balabandian et al. 2025). In cellular models, psilocin has been shown to reduce the production of reactive oxygen species (ROS) and nitric oxide (NO) in activated microglia, effects mediated by interaction with 5‐HT2 receptors (Wiens et al. 2024). Psilocin also decreases phagocytic activity and limits the release of pro‐inflammatory cytokines such as TNF‐α, contributing to modulation of neuroimmune responses associated with neuroinflammation (Wiens et al. 2024).

These findings are complemented by recent studies showing that psilocin regulates the expression of TNF‐α, IL‐10, and BDNF in activated microglia through activation of 5‐HT2A, 5‐HT2B, 5‐HT7, tropomyosin receptor kinase B (TrkB), and aryl hydrocarbon receptor (AhR) receptors (Laabi et al. 2025). This interaction promotes an anti‐inflammatory and neurotrophic microglial phenotype, further supporting its therapeutic potential in neuroinflammatory conditions (Laabi et al. 2025). Beyond inflammation, psilocin modulates the BDNF/TrkB‐AKT signaling pathway, normalizing mature BDNF levels and increasing TrkB expression in the prefrontal cortex (Ascone et al. 2026). Consistent with these findings, a placebo‐controlled clinical study in healthy individuals reported an acute reduction in TNF‐α and a sustained decrease in IL‐6 and C‐reactive protein (CRP) 1 week after psilocybin administration. These immune changes were associated with lower glutamate levels in the hippocampus and improvements in mood and social connectedness, highlighting the potential relevance of the anti‐inflammatory properties of psilocybin for treating disorders with an inflammatory component (Mason et al. 2023). In line with this, a recent preclinical study demonstrated that psilocybin significantly reduced the expression of IL‐1β, IL‐6, TNF‐α, monocyte chemoattractant protein‐1 (MCP‐1), and cyclooxygenase‐2 (COX‐2) in an lipopolysaccharide‐induced liver inflammation model in mice, with post‐treatment administration showing particularly robust effects. Histological analyses confirmed reduced inflammatory infiltration and improved tissue morphology, supporting its potential in treating acute and chronic liver inflammation (G. I. Robinson et al. 2025). These anti‐inflammatory and neuroprotective effects position psilocin as a promising candidate for therapeutic interventions in disorders involving seizures and brain inflammation (Balabandian et al. 2025; Wiens et al. 2024).

Preclinical models have shown that psilocin mitigates neurodegenerative processes under conditions of oxidative stress and neuroinflammation by significantly reducing ROS and NO production in activated microglial cells. This anti‐inflammatory effect appears to be mediated by activation of 5‐HT2A receptors, as it is reversed with specific antagonists (Wiens et al. 2024). These findings support the therapeutic potential of psilocin in neurodegenerative diseases associated with chronic inflammatory processes, such as Alzheimer's disease and other neurodegenerative disorders, as well as in treatment‐resistant depression (Wiens et al. 2024; Zheng et al. 2024). Emerging evidence also suggests that psilocybin may hold promise for traumatic brain injury (TBI), due to its anti‐inflammatory effects, potential to promote neurogenesis and synaptogenesis, and relevance for managing post‐TBI depression, though further clinical research is needed to establish safety and dosing protocols (Palmer et al. 2025).

In addition to the neuroprotective impact observed in preclinical models, clinical case reports have shown promising outcomes in complex neurological conditions. For example, a patient with complex regional pain syndrome (CRPS) refractory to multiple conventional treatments, including spinal cord stimulation and ketamine, experienced sustained pain reduction and functional improvement following psilocybin‐assisted sessions combined with conventional therapy (Jevotovsky et al. 2024). This sustained analgesic effect is associated with neuroplasticity mechanisms mediated by 5‐HT2A receptors, with implications for central pain modulation. In a related context, a recent study reported that psilocybin may enhance neurogenesis through 5‐HT2A receptor activation, suggesting potential therapeutic benefits for disorders involving impaired neuroplasticity such as anorexia nervosa (Downey et al. 2024). This situates the discussion within broader considerations regarding the ethical implementation of psychedelics in pain management, particularly in conditions such as CRPS, where traditional therapies often fail (C. L. Robinson et al. 2024).

### 4.2 Emerging Clinical Applications

Psilocybin has also shown potential in the treatment of alcohol use disorder, particularly by interfering with the reconsolidation of alcohol‐related memories. In animal models, administration of psilocybin following memory reactivation significantly reduced alcohol‐seeking behavior, suggesting a therapeutic approach to prevent relapse. This effect is not linked to a direct reduction in consumption but rather to a disruption of memory processes, opening the possibility of combining psilocybin with techniques that reactivate consumption‐related memories to interrupt compulsive seeking patterns (Benvenuti et al. 2023). In support of this, psilocin has been shown to restore cortical homeostasis and excitatory/inhibitory balance in rodent models of methamphetamine‐induced neural dysregulation, suggesting a broader potential to modulate dysfunctional brain circuits involved in compulsive behaviors (Wang et al. 2023).

Additionally, an open‐label pilot study evaluated the feasibility, safety, and preliminary efficacy of integrating three psilocybin‐assisted therapy sessions into an evidence‐based smoking cessation protocol in 15 adult smokers. Participants received weekly cognitive behavioral therapy during the first 4 weeks, followed by three psilocybin sessions in weeks 5, 7, and 13, with escalating doses (20 mg/70 kg in the first session and 30 mg/70 kg in the subsequent two). Results showed a 7‐day abstinence rate of 80% at the end of treatment, biochemically verified, and a substantial proportion of participants remained tobacco‐free more than 1 year after the intervention (Johnson et al. 2014). These findings reinforce the therapeutic potential of psilocybin beyond the depressive spectrum, opening new lines of clinical research. Similarly, recent evidence indicates that psilocybin‐assisted treatment is a safe and potentially effective intervention for post‐treatment Lyme disease syndrome (PTLD). In a pilot study, participants reported a 40% reduction in general symptom burden and a 13% improvement in quality of life 6 months after treatment, with significant gains in mood, fatigue, and pain. No serious adverse events were reported, suggesting that psilocybin could be a promising option for PTLD and other chronic infection‐related conditions (Garcia‐Romeu et al. 2026). In this context, some studies are exploring the development of psilocybin analogs that retain therapeutic potential but lack or significantly reduce psychedelic effects (Raithatha et al. 2024). To support these advancements, recent research has optimized de novo psilocybin synthesis in recombinant *Escherichia coli* systems. Initial metabolic engineering achieved a production of 79.4 mg/L (Huang et al. 2025), while further multi‐species gene optimization, specifically utilizing the psiM gene from *Gymnopilus dilepis*, has pushed this limit to a record 1.46 g/L. This efficient biosynthetic route provides a cost‐effective platform for pharmaceutical‐grade psilocybin (Keller et al. 2025). This establishes a sustainable platform for the industrial manufacture of psilocybin and its modified therapeutic analogs.Emotional resilience and the capacity to cope with traumatic experiences have also been observed, effects particularly relevant for the clinical management of post‐traumatic disorders (Gattuso et al. 2024; Strauss et al. 2022). In line with this, recent studies have reported sustained reductions in symptoms of post‐traumatic stress disorder (PTSD), treatment‐resistant depression, and trauma‐related chronic pain, further supporting the broad therapeutic potential of psilocybin‐assisted psychotherapy (Gattuso et al. 2024; Marcinkowski et al. 2024). Indeed, most clinical trials and systematic reviews agree that controlled psilocybin use within structured psychotherapeutic protocols can induce significant and lasting improvements in patients diagnosed with major depression (Gattuso et al. 2024; Menon et al. 2025; Meccia et al. 2023; Nichols 2020; Shah et al. 2025), including patients with terminal cancer (Bellman 2024). This is particularly relevant for cases of treatment‐resistant depression, where psychedelic‐assisted therapy has emerged as a promising alternative supported by clinicians and preliminary clinical outcomes (Lapid et al. 2025; Negrine et al. 2024).

Expanding upon these psychiatric indications, eating disorders have recently surfaced as a major emerging clinical application for psilocybin‐assisted therapy. Recent clinical evidence demonstrates that psilocybin therapy is safe and feasible for adult females with anorexia nervosa. Specifically, this intervention has shown potential in reducing the psychological rigidity and severe behavioral patterns characteristic of the disorder, offering a promising alternative for psychiatric conditions that are traditionally highly resistant to conventional treatments (Douglass et al. 2026).

Recent experimental evidence indicates that psilocybin, in addition to its psychiatric and neurological applications, may also exert systemic geroprotective effects (Kato et al. 2025). In vitro, psilocin extended cellular lifespan by up to 57% in human fibroblasts through delayed senescence, preservation of telomere length, upregulation of SIRT1 expression, reduction of oxidative stress via both decreased Nox4 and increased Nrf2 activity, and downregulation of cell‐cycle arrest markers (p21, p16). Complementing these findings, chronic administration of low‐dose psilocybin (0.05 mg/kg) in mouse models of metabolic stress (high‐fat and high‐fructose diet) has been shown to normalize the hepatic lipidome and restore insulin signaling through increased serine/threonine kinase 1 (Akt1) phosphorylation. These metabolic improvements are largely driven by 5‐HT2B receptor antagonism in the liver, which reduces lipid accumulation and enhances leptin sensitivity in both hepatic and skeletal muscle tissues (Colognesi et al. 2026). Notably, while 5‐HT2B antagonism is central to these effects, the multifaceted pharmacology of psilocin (including partial agonism at 5‐HT2C receptors) further supports mitochondrial biogenesis and prevents muscle atrophy (Colognesi et al. 2026). In vivo, monthly administration of psilocybin in aged mice increased survival rates from 50% to 80% over a 10‐month period, accompanied by phenotypic improvements in fur quality. These findings suggest that the potential long‐lasting therapeutic benefits of psilocybin may be mediated by its influence on fundamental pathways of biological aging and metabolic homeostasis (Colognesi et al. 2026; Kato et al. 2025).

### 4.3 Neuroplasticity, Reconnection of Neural Circuits, and Desynchronization

Psilocin induces an increase in metabolic activity in the prefrontal cortex, and anterior cingulate cortex, facilitating states of synaptic plasticity that promote the restructuring of neural networks (Vejmola et al. 2021; Madsen et al. 2021; Nichols 2020). This process is accompanied by increased functional connectivity between key areas such as the visual cortex, prefrontal cortex, cingulate cortex, temporal cortex and bilateral anterior cortex, favoring the integration of sensory, emotional, and cognitive information (Madsen et al. 2021). Recent evidence indicates that a single dose of psilocybin induces a rapid structural remodeling of dendritic spines in the medial frontal cortex (Jiang et al. 2026).

Additionally, neuroimaging studies have shown that psilocin facilitates the synchronization of neural circuits during introspective, meditative, and transformative subjective experiences, which is associated with greater emotional coping capacity and reduced symptoms related to mood disorders (Nichols 2020; Madsen et al. 2021). In particular, the initial disintegration and subsequent reconfiguration of connectivity in the default mode network (DMN) appear to be linked to a deep restructuring of cognitive patterns, facilitating the reevaluation of self‐related concepts, emotional re‐significant, and the reduction of intrusive thoughts (Deco et al. 2024; Sharma et al. 2023; Strauss et al. 2022). Consistent with this, changes in resting‐state functional connectivity and brain modularity can persist for up to 1 week following a single dose, suggesting a prolonged impact on large‐scale network reorganization (Purple et al. 2025). This supports recent clinical studies proposing that psychological flexibility and experiential acceptance could act as key mechanisms in the sustained therapeutic effects of psilocybin, especially in patients with treatment‐resistant major depression (Sloshower et al. 2024).

The increase in metabolic activity is attributed to the ability of psilocin to enhance cerebral blood flow and oxygen demand in regions such as the frontal cortex, anterior cingulate cortex, and amygdala, resulting in increased neuronal activity and glucose metabolic rate (Nichols 2020; Sharma et al. 2023; Spain et al. 2015). However, this increase may also result from alterations in neurovascular coupling (Spain et al. 2015).

Clinical studies have demonstrated that plasma levels of psilocin and 5‐HT2A receptor occupancy in the brain are closely correlated with the subjective intensity of the psychedelic experience, supporting the functional involvement of these receptors in the acute effects of psilocin (Madsen et al. 2019). Additionally, recent neuroimaging research has shown that psilocin plasma concentration is associated with a reduction in frontoparietal functional connectivity, helping to characterize acute psychedelic states and potentially guiding the development of imaging‐based measures to assess their clinical impact (Olsen et al. 2022).

Moreover, profound psychedelic experiences facilitated by psilocybin have been observed to induce lasting changes in personality, particularly an increase in the trait of openness, as well as in prosocial, introspective, and altruistic behaviors, even 1 year after administration (Orzechowski et al. 2025). These sustained effects may be mediated by neuronal plasticity and functional reconnection within the DMN, consolidating more flexible and adaptive cognitive patterns (Sharma et al. 2023). The proposed mechanisms of action for psilocybin/psilocin, as well as their potential clinical applications and identified risks, are summarized in Table 1.

**TABLE 1:** Summary of pharmacological mechanisms and clinical implications of psilocybin/psilocin.

Reference	Pharmacological mechanism	Therapeutic application/clinical relevance
Afrooghe et al. (2025)	Agonist of 5‐HT2A receptors in non‐neuronal tissue; interference with the kynurenine pathway	Antipruritic effect
Alyahya and Al Saleem (2025)	N/A	Systematic review on its therapeutic use in depression, anxiety, obsessive‐compulsive disorder, and addiction; potential risk of psychosis
Ascone et al. (2026)	Modulation of the brain‐derived neurotrophic factor (BDNF)/tropomyosin receptor kinase B (TrkB)‐protein kinase B (AKT) signaling pathway	Therapeutic potential of psilocybin microdosing as a strategy to improve cognitive function and alleviate neurodevelopmental impairments in conditions such as fragile X syndrome and autism spectrum disorder
Balabandian et al. (2025)	Agonist of 5‐HT1A receptor and modulation of the endocannabinoid system, involving the nitrergic, opioid, kynurenine, and cyclic guanosine monophosphate pathways	Anticonvulsant effect in epilepsy models; therapeutic potential for disorders involving neuronal hyperexcitability
Bysiek et al. (2025)	Agonist of 5‐HT receptors, modulation of neurotransmitter release dopamine, noradrenaline, serotonin, glutamate, GABA and acetylcholine, and regulation of the hypothalamic‐pituitary‐adrenal axis.	Treatment of depression, anxiety, obsessive‐compulsive disorder and substance addiction.
Benvenuti et al. (2023)	Agonist of multiple 5‐HT receptors, especially 5‐HT2A and 5‐HT1A	Potential clinical application in relapse prevention for alcohol use disorder by interfering with memory reconsolidation
Brito‐da‐Costa et al. (2023)	Inhibition of CYP450 isoforms	Potential risk of pharmacological interactions due to hepatic enzyme inhibition
Colognesi et al. (2026)	Antagonism of 5‐HT2B, partial agonism of 5‐HT2A and 5‐HT2C	Improvement of hepatic lipid profile and insulin sensitivity. Possible contribution to leptin sensitization and improvement of muscle function.
Dawood‐Hristova and Pérez‐Jover (2023)	N/A	Therapeutic alternative for treatment‐resistant depression with sustained antidepressant effects lasting up to 12 months
Deco et al. (2024)	Agonist of 5‐HT2A receptors; induces top‐down reorganization of brain dynamics in patients with depression	Comparable efficacy to escitalopram in treatment‐resistant depression
Downey et al. (2024)	Neurogenesis mediated by 5‐HT2A receptor activation	Potential application in the treatment of anorexia nervosa
Effinger et al. (2024)	Significant increase in c‐Fos expression in the paraventricular nucleus (PVN) of the hypothalamus	Potential therapeutic implication in stress‐related behavioral modulation; relevance of the PVN in psychedelic effects
Garcia‐Romeu et al. (2026)	N/A	Improved quality of life and reduced fatigue and pain in patients with post‐treatment Lyme disease syndrome (PTLD).
Hakami et al. (2023)	Psilocybin exhibits higher affinity for 5‐HT2A receptors than serotonin	Supports rational design of antidepressants based on the structure of psilocin and its receptor affinity
Jevotovsky et al. (2024)	Agonist of 5‐HT2A receptors; possible central modulation of sensitization and plasticity in neuropathic pain	Chronic pain management
Johnson et al. (2014)	N/A	Psilocybin may serve as an adjunct to smoking cessation by enhancing self‐efficacy and shifting life priorities
Kato et al. (2025)	Modulation of several pathways associated with cellular aging, including increased sirtuin1 (SIRT1) expression, reduced markers of cell cycle arrest (p21, p16), elevated markers of proliferation (PCNA) and DNA replication (pRB), and decreased oxidative stress	Potential geroprotective agent: Extension of cellular lifespan in vitro and increased survival in aged mice; possible impact on multiple age‐related diseases and healthy aging
Kimberly et al. (2024)	Partial agonist of 5‐HT2A receptors, weak serotonin transporter inhibitor, and competitive monoamine oxidase (MAO) inhibitor	Reduction in the subjective intensity of psychedelic effects when combined with selective serotonin reuptake inhibitors (SSRIs); theoretical risk of serotonergic toxicity
Laabi et al. (2025)	Regulation of TNF‐α and brain‐derived neurotrophic factor (BDNF) via 5‐HT2A, 5‐HT2B, 5‐HT7, tropomyosin receptor kinase B (TrkB), and hydrocarbon receptor (AhR) signaling; promotion of an anti‐inflammatory and neuroprotective microglial phenotype	Therapeutic potential for neuropsychiatric conditions involving neuroinflammation; supports a shift toward an immunomodulatory microglial state
Madsen et al. (2019)	Dose‐dependent 5‐HT2A receptor occupancy correlated with plasma psilocin levels	Direct correlation between plasma psilocin levels and 5‐HT2A receptor occupancy; relevant for dose‐response studies
Madsen et al. (2021)	Plasma psilocin level correlates with reduced integrity of brain networks, including the default mode network (DMN), and reduced network segregation	N/A
Marcinkowski et al. (2024)	Agonist of 5‐HT2A receptors	Reduction of symptoms in post‐traumatic stress disorder (PTSD), depression, and chronic pain
Mason et al. (2023)	Acute reduction of TNF‐α and persistent decrease of IL‐6 and C‐reactive protein (CRP); correlated with lower glutamate levels in the hippocampus	Sustained improvement in mood and social connectedness; potential therapeutic relevance for inflammation‐related disorders
Meccia et al. (2023)	Acute and sustained modulation of connectivity in the medial prefrontal cortex; effect associated with structural neuroplasticity	Long‐lasting therapeutic effects in major depression and functional neural connectivity
Menon et al. (2025)	Agonist of 5‐HT2A receptor‐mediated downstream signaling processes; increased cortical neuroplasticity	Reduction of major depressive symptoms
Modzelewski et al. 2025; Oblak et al. 2024; Pop and Dinkelacker 2024; Polito and Stevenson 2019; Sanz et al. 2022.	N/A	In microdosing contexts, reported effects include increased creativity, cognitive flexibility, and perceived authenticity, along with reductions in depressive and stress‐related symptoms. Mild adverse effects such as anxiety, cognitive disorganization, or psychotic‐like symptoms have also been documented. Findings stem from studies including psilocybin among other classic psychedelics
Neumann et al. (2024)	Agonist of 5‐HT4 receptors in cardiac tissue	Risk of cardiovascular effects
Negrine et al. (2024)	N/A	Psychedelic‐assisted therapy for treatment‐resistant depression
Olsen et al. (2022)	Plasma psilocin level‐dependent reduction in frontoparietal functional connectivity	Supports the use of functional connectivity as a correlate of psilocin plasma levels and may inform future development of imaging‐based tools to monitor acute psychedelic states
Orzechowski et al. (2025)	Agonist of 5‐HT2A receptors with modulation of glutamatergic and dopaminergic pathways	Therapeutic effects on mood. Reported risks include transient anxiety, mild hypertension, and emotional lability
Palmer et al. (2025)	Reduction of multiple pro‐inflammatory cytokines—specifically TNF‐α, interferon‐gamma (IFN‐γ), inyterleukin‐6 (IL‐6), and interleukin‐8 (IL‐8)—enhancement of dendritic spine density and promotion of neuroplasticity	Potential therapeutic application in traumatic brain injury (TBI)
Pawlicki et al. (2024)	Agonist of 5‐HT2A receptors; reduction in brain activity in regions implicated in depression	Long‐lasting reduction of depression and anxiety with a low adverse effect profile
Purple et al. (2025)	Acute reduction of neuronal firing and complexity in the medial prefrontal cortex, followed by a long‐term decrease in network modularity.	Improvement of depressive symptoms and emotional processing in treatment‐resistant depression.
Raithatha et al. (2024)	N/A	Developed of psilocin prodrugs with similar therapeutic benefits but a reduced duration of psychedelic effects compared with psilocybin.
C. L. Robinson et al. (2024)	Activation of 5‐HT2A receptors, potentially leading to neuroplasticity involved in chronic pain modulation	Potential therapeutic application in chronic pain
G. I. Robinson et al. (2025)	Reduction of pro‐inflammatory cytokine expression, including IL‐1β, IL‐6, TNF‐α, and monocyte chemoattractant protein‐1 (MCP‐1), as well as cyclooxygenase‐2 (COX‐2) in a lipopolysaccharide‐induced liver inflammation model	Potential therapeutic application in acute and chronic inflammatory liver diseases; possible use as a complementary systemic anti‐inflammatory agent
Sarparast et al. (2022)	Agonist of 5‐HT2A receptors and modulation of serotonergic neurotransmission	Potential pharmacodynamic interactions with antidepressants; theoretical risk of serotonergic toxicity
Shah et al. (2025)	Agonist of 5‐HT2A receptors and glutamatergic mGlu2 receptors	Neuroplasticity and reduction of depression and anxiety through facilitation of emotional processing
Sharma et al. (2023)	Interacts with multiple serotonin receptor subtypes, including 5‐HT1A, 5‐HT1D, 5‐HT2A, 5‐HT2B, and 5‐HT2C; higher affinity for presynaptic 5‐HT2A receptors	Evidence for therapeutic use in depression, anxiety, and alcohol use disorders; rare but reported risk of suicidal ideation and self‐injury.
Shao et al. (2021)	Agonist of 5‐HT2A receptors; increases spine density and size in frontal cortical pyramidal neurons, accompanied by elevated excitatory activity	Preclinical evidence of sustained antidepressant effects associated with synaptic plasticity and restoration of stress‐affected neural circuits
Singer et al. (2024)	N/A	Positive derealization; when combined with meditation, it enhances insightfulness
Sloshower et al. (2024)	N/A	Improvement in psychological flexibility and experiential acceptance was associated with reduced depression.
Spain et al. (2015)	Agonist of 5‐HT2A receptors in the prefrontal and anterior cingulate cortex; neurovascular modulations independent of neuronal activity	Alteration of neurovascular coupling and activity in cortical regions involved in cognitive functions
Strauss et al. (2022)	Agonist of 5‐HT2A receptors; increases cortical activity via postsynaptic glutamate‐mediated effects	Therapeutic potential in psychiatric and neurological disorders
Thomas et al. (2022)	N/A	Acute and transient alteration in the onset and continuity of rapid eye movement (REM) and non‐rapid eye movement (NREM) sleep stages
Vejmola et al. (2021)	Desynchronization of neural networks; reduced functional connectivity in the frontal and sensorimotor cortex	N/A
Viohl et al. (2025)	Agonism at 5‐HT2A receptors coupled to Gαq proteins; binding to neurotrophic TrkB and stimulation of endogenous BDNF.	Molecular basis for antidepressant effects
Wang et al. (2023)	Altered expression of dopamine D2 receptor (D2R) and phosphorylated extracellular signal‐regulated kinase in the prefrontal cortex, nucleus accumbens, and ventral tegmental area; 5‐HT2A receptor agonist	Inhibition of methamphetamine‐conditioned place preference; potential application in addiction treatment
Wiens et al. (2024)	Microglial modulation and reduction of reactive oxygen species and nitric oxide; effects mediated by 5‐HT2A receptor activation	Therapeutic potential in neurodegenerative diseases associated with neuroinflammation
Zheng et al. (2024)	Agonism at 5‐HT2A, 5‐HT1A, and 5‐HT2C receptors	Stimulation of neuroplasticity, reduction of inflammation, and relief of anxiety and depression in Alzheimer's disease patients

Abbreviation: N/A, Not applicable.

## 5 Microdosing of Psilocybin

In parallel with the growing interest in the clinical use of full‐dose psilocybin assisted by psychotherapy, a widely spread contemporary phenomenon known as microdosing has emerged. This approach involves the repeated administration of subperceptual doses of psilocybin, usually less than 10% of a full psychedelic dose, with the aim of achieving cognitive, emotional, or creative benefits without inducing evident perceptual alterations (Cavanna et al. 2022; Modzelewski et al. 2025).

However, despite the popularization of this practice, systematic research with larger samples and controlled designs is still required to accurately characterize its efficacy and safety. Crucially, systematic studies warn that microdosing effects may be partly attributable to expectancy and placebo mechanisms. For example, Polito and Stevenson (2019) found that prior beliefs about the positive effects of microdosing did not fully align with the outcomes observed. Furthermore, in a double‐blind study using standardized amounts of *P. cubensis*, Cavanna et al. (2022) found no consistent improvements in creativity or cognitive function, although significant alterations in brain electrical activity were observed (decreased theta band in electroencephalogram). This suggests that some subjective effects may not be supported by measurable objective changes.

Bearing these preliminary observations and methodological caveats in mind, various observational and self‐report studies have documented perceived positive effects on mood, productivity, focus, and creativity. Longitudinal studies such as that of Polito and Stevenson (2019) reported improvements in measures of depression and attention during microdosing regimens, though they also observed an increase in neuroticism, suggesting mixed effects. Similarly, Pop and Dinkelacker (2024) reported a greater sense of subjective authenticity on microdosing days, associated with an increase in the number of daily activities performed and their associated satisfaction, which could contribute to a better perception of overall well‐being.

Beyond these human observational outcomes, recent preclinical research has begun to unravel the precise neurobiological pathways behind the cognitive effects of microdosing. Specifically, Ascone et al. (2026) demonstrated that a 2‐week psilocybin microdosing regimen successfully rescued object recognition memory deficits in a rat model of Fragile X Syndrome and Autism Spectrum Disorder. Crucially, their pharmacological experiments revealed that this therapeutic effect persists even when classical serotonergic receptors (5‐HT2A and 5‐HT1A) are blocked but is completely abolished upon inhibiting the TrkB receptor. At the molecular level, this microdosing protocol normalized mature BDNF levels and restored downstream AKT signaling in the prefrontal cortex, highlighting the activation of neurotrophic pathways rather than direct serotonergic receptor stimulation as the key driver for cognitive enhancement.

A recent double‐blind, placebo‐controlled study using 0.5 g of *Psilocybe cubensis* (*P. cubensis*) found statistically significant differences in the spontaneous speech of the participants following microdosing. Higher levels of verbosity and positive emotional content were recorded, particularly in the dimensions of perception, mood, and alertness, suggesting subtle but identifiable effects through natural language analysis (Sanz et al. 2022).

However, it has been documented that the psilocybin concentration can vary widely between and even within strains of *P. cubensis*, due to genetic, environmental, and cultivation‐related factors. This variability makes the use of fixed mushroom weight as a dose reference pharmacologically imprecise, limiting standardization in controlled studies (Kurzbaum et al. 2025).

To address this variability, recent clinical protocols have utilized standardized doses of pure psilocybin. For instance, a titration study for psychological distress in palliative care implemented a 3‐week regimen, starting at 1 mg daily and escalating to 3 mg based on participant tolerance. This intervention proved to be a safe and feasible strategy for patients with advanced illness, resulting in significant improvements in depression, anxiety, and demoralization compared to baseline (Downar et al. 2026). Such findings suggest that standardized microdosing could be a scalable option for the urgent need for treatments in palliative settings, although larger placebo‐controlled trials are required to confirm these outcomes. Complementarily, a qualitative study conducted in Slovenia with participants who self‐administered psilocybin microdoses reported perceived improvements in creativity, mindfulness, and cognitive flexibility. However, negative experiences were also reported, including confusion, sensory overload, and reduced cognitive stability, particularly in unstructured work settings. These findings underscore the importance of “set and setting” and therapeutic support in evaluating microdosing outcomes (Oblak et al. 2024).

Nevertheless, despite its current speculative nature, this practice continues to represent an emerging line of research potentially relevant for the development of low‐intensity psychedelic interventions that retain some therapeutic benefits without inducing altered states of consciousness.

## 6 Conclusions

Psilocybin emerges as a promising compound with multimodal therapeutic effects, supported by converging evidence from molecular, preclinical, and clinical studies. Its interaction with serotonergic receptors, particularly 5‐HT2A, initiates complex neuroplastic, anti‐inflammatory, and neuromodulatory cascades that support emotional regulation, cognitive restructuring, and the restoration of brain network integrity. Beyond its antidepressant and anxiolytic effects, psilocybin demonstrates a diverse array of pharmacological mechanisms of action, reinforcing its potential in the treatment of conditions such as addiction, neuropathic pain, and neuroinflammation.

Nonetheless, its therapeutic application requires thoughtful clinical integration. Factors such as individual variability, long‐term effects, and ethical considerations remain central to its responsible use. Continued interdisciplinary research, regulatory innovation, and equitable access will be essential to realize its full potential. The future of psilocybin in medicine depends not only on its biological efficacy, but also on our capacity to approach its integration with scientific rigor, cultural awareness, and therapeutic precision.

## Funding

The author has nothing to report.

## Ethics Statement

The author has nothing to report.

## Consent

The author confirms consent to publish.

## Conflicts of Interest

The author declares no conflicts of interest.

## Data Availability

The author has nothing to report.
